# Biocomposites Based on Biopolyamide with Reduced Water Absorption and Increased Fatigue Strength

**DOI:** 10.3390/polym17111559

**Published:** 2025-06-03

**Authors:** Patrycja Bazan, Elisabeth Egholm Jacobsen, Anna Olsen, Kristofer Gunnar Paso

**Affiliations:** 1 Faculty of Material Engineering and Physics, Cracow University of Technology, 31-155 Krakow, Poland; patrycja.bazan@pk.edu.pl; 2 Department of Chemistry, Norwegian University of Science and Technology, 7491 Trondheim, Norway; 3 Department of Mechanical and Industrial Engineering, Norwegian University of Science and Technology, 7491 Trondheim, Norway; anna.olsen@ntnu.no; 4 Department of Chemical Engineering, Norwegian University of Science and Technology, 7491 Trondheim, Norway; kristofer.g.paso@ntnu.no

**Keywords:** microsilica, reinforcing fibers, hybrid polymer composites, fatigue strength, water absorption

## Abstract

In this study, composites were developed using a biopolyamide matrix modified with microsilica at varying concentrations (0.5–2% by weight). These composites underwent water absorption analysis, and diffusion velocity was assessed. Based on the findings, hybrid composites incorporating aramid, basalt, and carbon fibers, further modified with 2% microsilica by weight, were fabricated. Investigations into fundamental mechanical properties, microstructure analysis, and accelerated fatigue tests were conducted. The results demonstrate that microsilica positively influences the enhancement of fatigue strength and mechanical properties of the composites. Specifically, microsilica is found to increase the approximate fatigue strength by 15% for the base material modified with 2 wt.% microsilica, by approximately 5% for composites with aramid fiber, and by between 10 and 15% for composites with basalt and carbon fiber. Furthermore, the incorporation of microsilica reduces water absorption in polymer composites, potentially enhancing their durability in humid environments and increasing resistance to degradation.

## 1. Introduction

Polyamide (PA) is an engineering material distinguished by its high strength properties while maintaining excellent impact resistance. Additionally, it serves as an electrical insulator and exhibits resistance to elevated temperatures, along with superior tribological properties. Consequently, it finds applications in the automotive, textile, machinery, construction, and packaging industries, as well as in electronics and electrical engineering [1]. Currently, there is a growing interest in bio-based polymers as a viable alternative to their petrochemical counterparts [2,3]. Biopolyamide derived from biological sources is predominantly synthesized from castor oil [4]. Research on biopolyamide-based composites demonstrates that synthetic, mineral, and natural fibers can effectively serve as reinforcing agents. Notable examples include carbon, glass, and cellulose fibers [5,6,7]. Aramid fibers exhibit exceptional resistance to abrasion, fatigue, creep, temperatures, and chemicals due to their compact molecular structure and crystallinity. Kevlar 49 is one of the main types used in structural applications. During extrusion, Kevlar forms straight polymer chains parallel to the fiber axis, with hydrogen bonds between adjacent molecules stabilizing the structure. This creates a highly anisotropic fiber with superior strength and modulus longitudinally versus radially [8,9,10]. Biopolyamide–aramid composites are extensively utilized, particularly in the automotive sectors, where materials that are both lightweight and possess high strength are essential. Owing to their enhanced mechanical properties, these composites are employed in the manufacture of structural components, protective clothing, and safety equipment [11]. An illustrative example is the incorporation of aramid fibers into biopolyamide matrices for the fabrication of bulletproof vests and helmets, which enhances their impact resistance and durability [12,13]. The incorporation of aramid fiber resulted in an approximate 15% enhancement in tensile strength, while the Young’s modulus exhibited a substantial increase of nearly 40% compared to the base material. Experimental findings were evaluated against modeling parameters. Additionally, thermal analysis was conducted to assess the impact of the fibers on the degree of crystallinity and melting temperature [3,14].

Carbon fibers are high-performance materials with carbon content exceeding 90%. They combine ultra-lightweight properties with superior mechanical characteristics in their specific strength and modulus. Their dimensional stability, corrosion resistance, and low density make them ideal for aerospace, automotive, and transport applications. Alignment of graphite-like crystallites and graphitization degree are key factors in their mechanical performance and polymer matrix reinforcement [15,16]. Empirical evidence indicates that the incorporation of carbon fibers substantially augments the tensile strength, flexural stiffness, and overall rigidity of the composite material. For instance, polyamide 10.10 composites with up to 40% carbon fiber by weight demonstrate a tensile strength of approximately 185 MPa and a modulus of elasticity surpassing 23 GPa [6]. Biopolyamide–carbon composites are extensively utilized across various industries. In the automotive sector, these composites are employed in the production of lightweight components, thereby enhancing the energy efficiency of vehicles. Their superior strength-to-weight ratio renders them particularly suitable for semi-structural structures and components, facilitating weight reduction without compromising safety [17]. The integration of biopolymers and carbon fibers in the aerospace sector and sports equipment manufacturing facilitates the creation of components that exhibit both high strength and flexibility. Furthermore, the fatigue resistance and thermal stability of these composites render them suitable for use in components subjected to severe environmental conditions and mechanical stresses [16,18,19].

Basalt fibers are distinguished by their superior mechanical properties, including a high tensile strength of up to 4800 MPa and notable thermal stability, rendering them suitable for high-performance applications. The primary constituent of these fibers is silicon dioxide, which enhances their strength, chemical stability, and elasticity [20,21]. Basalt fiber production involves melting volcanic rock and spinning at high temperatures, yielding minimal thermal expansion and superior acoustic insulation. Basalt fiber shows economically advantageous strength compared to costlier carbon and glass fibers. As an inorganic material, it exhibits high elastic modulus, strength, temperature resistance, dimensional stability, and chemical resistance, while being non-toxic and sustainable. With diameters of 10–20 μm, basalt fibers demonstrate better thermal properties than glass fibers, withstanding temperatures of 1100–1200 °C without damage, making them comparable to glass and carbon fiber [20,22]. The incorporation of basalt fibers into biopolyamides significantly enhances the flexural strength and elastic modulus of composites, thereby facilitating more efficient stress transfer and augmenting load-bearing capacity [23,24]. Basalt reinforcement composites exhibit considerable versatility and are employed across a diverse array of industries, including the automotive, construction, and aerospace industries. Within the automotive sector, their lightweight properties and high mechanical strength significantly enhance the energy efficiency of vehicles [25]. In the field of aerospace, basalt fibers are particularly well suited for components subjected to extreme temperature variations and mechanical stresses during flight, owing to their high resistance to heat [26]. Given that the base material is a condensation polymer prone to water absorption that reduces mechanical properties, and fiber incorporation increases absorption, this research aims to develop composites with reduced water absorption and enhanced fatigue strength. Microsilica’s extensive surface area allows it to occupy matrix voids, improving interfacial adhesion between polymer chains and creating a compact structure. Studies show that microsilica creates interfacial surfaces that restrict moisture migration through the material [27]. Reducing the porosity of the composite can enhance its microstructure, thereby decreasing water penetration and improving mechanical properties such as tensile strength [28]. The pozzolanic activity of microsilica creates bonding sites within the polymer matrix, enhancing water resistance. This reduces hydrophilic polymer swelling in humid environments, improving durability. Its interaction with polymer components forms a network that reduces water absorption while maintaining flexibility [27,29].

In the presented research, fundamental investigations into the physical properties, absorption mechanisms, and mechanical and fatigue characteristics of biopolyamide-based composites modified with aramid, basalt, carbon, and microsilica fibers were conducted. These studies facilitated the verification of hypotheses concerning the development of advanced biocomposites with reduced water absorption. Such materials present a viable alternative to traditionally utilized polyamide composites reinforced with glass fiber. It was posited that microsilica, as a mineral material, does not absorb water and, by filling voids within the composite’s microstructure, should further diminish water absorption. This results in enhanced durability in humid environments and improved resistance to degradation. A novel aspect of this work is the demonstration that microsilica not only enhances mechanical properties but also reduces moisture penetration. Furthermore, the composites were subjected to identical fatigue conditions, enabling a direct comparison of their properties and insights into the impact of microsilica on individual materials and their behavior under variable loads. This methodology significantly advances the field of modern polymer composites. A comprehensive research approach has facilitated the development of innovative, highly durable polymer composites, characterized by the strategic selection of sustainable raw materials.

## 2. Materials and Methods

### 2.1. Materials

During the experimental phase of this study, sixteen materials were made. The employed matrix was Biopolyamide EcoPaXX^®^ Q150-D PA410 (DSM Plastic, Heerlen, The Netherlands). Microsilica was incorporated as a modifier (white microsilica G95, Mikrosilika Trade, Stalowa Wola, Poland). As a reinforcement, basalt fiber (BF) (KV02M chopped strand series with a diameter of 13 µm and a length of approximately 3.2 mm, Kemenny Vek, Dubna, Russia), aramid fiber (AF) (Para-Aramid with a diameter of 13 µm and a length of approximately 3 mm, Rocket-Fibers, Hatfield, PA, USA), and chopped carbon fiber (CF) with a diameter of 10–15 µm and a length of approximately 6 mm (Rocket-Fibers, Hatfield, PA, USA) were used. The test specimens were manufactured through injection molding. The processing parameters are detailed in Table 1. Prior to the injection process, the biopolyamide and additives underwent a drying procedure for 24 h at 80 °C. The sample production process was carried out without prior compounding of the components. Dry composite components were weighed in appropriate proportions, mixed, and poured into the injection molding machine hopper. A description of the produced materials is provided in Table 2.

### 2.2. Methods of Testing

Water absorption in the “through the thickness layer” direction of solid materials was assessed in accordance with ISO 62:2008 [30]. The measurement of water absorption was conducted using the standard method. Initially, the samples were weighed with a precision of 0.001 g and subsequently immersed in a vessel containing water at a temperature of 23 °C. The samples were incubated in water for 1, 2, 3, 7, 14, 21, 28, and 90 days. After each incubation period, the samples were removed from the water, dried with tissue paper, and reweighed. The results were expressed in terms of weight absorbability Equation (1):

(1) $$W=\frac{W_{n}-W_{0}}{W_{0}}\cdot 100\%,$$

where

W—water absorption (%);
$W_{n}$—weight of the sample after incubation in water (g);
$W_{0}$—initial sample weight (g)

As part of the research, theoretical values of diffusion coefficients and kinetics were calculated [31,32]. Fick’s Diffusion Coefficient (D) was estimated using Equation (2) to the extent that the percentage of weight gain was less than 60% of the equilibrium value ($M_{m}$):

(2) $$D=\pi \cdot {\left(\frac{k\cdot h}{4M_{m}}\right)}^{2}$$

where

$M_{m}$—the maximum moisture content (g);
h—sample thickness (mm);
k—initial slope of curve M(t) as a function of $\sqrt{t}$ as shown in Equation (3):

(3) $$k=\frac{M_{2}-M_{1}}{\sqrt{t_{2}}-\sqrt{t_{1}}}$$

Diffusion behavior can be categorized based on the relative mobility of the penetrant and polymer segments. There are three distinct types: type I, or Fickian diffusion (Equation (2)), where the diffusion rate is significantly lower than the mobility of the polymer segment. In this scenario, equilibrium within the material is rapidly established and maintained over time. Type II diffusion is characterized by a higher rate of diffusion and penetrant mobility compared to other relaxation processes. This type is marked by the formation of a boundary between the swollen outer and inner polymer regions, which progresses at a constant rate until equilibrium is achieved throughout the entire polymer. Type III, or non-Fickian diffusion, also known as anomalous diffusion, occurs when the mobility of the penetrant and the relaxation of the polymer segments are comparable. This represents an intermediate behavior between type I and type II.

A distinction among these three mechanisms can be established through theoretical analysis of the absorption curve’s shape, which can be modeled using Equation (4):

(4) $$\frac{M_{t}}{M_{m}}=kt^{n},$$

where

$M_{t}$—moisture content (g);
$M_{m}$—saturation moisture content (g);
k and n—constant parameters.

The parameter n is related to the diffusion mode and takes different values, depending on the specific case: for Fick diffusion n = 0.5, type II n = 1, and for type III (anomalous diffusion) 0.5 < n < 1. The values of n and k can be determined from the slope and intercept of $M_{t}$/$M_{m}$ vs. t in a logarithmic plot obtained from the experimental data according to the Equation (5):

(5) $$\log\frac{M_{t}}{M_{m}}=\log\left(k\right)+n\log\left(t\right).$$

To ascertain diffusion coefficient and velocity, the water absorption method in boiling water, as specified by the PN-EN ISO 62:2000 standard [30], was employed. This method entails immersing the sample in boiling water for 30 ± 2 min, followed by cooling the sample in water at room temperature for 15 ± 1 min and subsequently weighing it. This procedure is repeated until saturation is achieved. Mechanical properties were evaluated using a static tensile test in accordance with PN-EN ISO 527-1:2010 [33]. A static tensile test was conducted on a Shimadzu AGS-X 10 kN testing machine (Kyoto, Japan) at a test speed of 10 mm/min. A three-point flexural test, in accordance with PN-EN ISO 178:2011 [34], was conducted using an MTS Criterion Model 43 universal testing machine (MTS System Corp., Eden Prairie, MN, USA). For impact testing, unnotched specimens were subjected to the Charpy method utilizing a Zwick/Roell HIT5.5P hammer (Ulm, Germany) in accordance with PN-EN ISO 179-1:2010 [35]. The results presented in this paper are the average value of the results obtained from at least three samples. Accelerated fatigue tests were conducted using an electrohydraulic servo fatigue testing machine, specifically the Shimadzu EHF-E Series (Kyoto, Japan). Standard strength samples were used for the tests as for the static tensile test according to ISO 3167:2003 [36], using dog bone-shaped samples. The research was grounded in the principles of the Lehr method, which was originally developed for fatigue testing of metals as an alternative to the conventional Wohler method for long-term testing [37]. The Lehr method is predicated on the observation that a notable increase in the dissipation energy, strain, and temperature of the tested samples precedes fatigue failure. Central to this method is the evaluation of these parameters during an accelerated fatigue test, characterized by cyclic loading with progressively increasing amplitude and their presentation in relation to the maximum stress. This methodology has proven effective in determining fatigue strength as a comparative parameter for assessing the fatigue properties of polymer composites with a thermoplastic matrix [38]. The accelerated fatigue parameters involved an initial load equivalent to 5% of the material’s tensile strength (140 N), with an incremental increase in amplitude by an additional 5% after 1000 cycles. The experiment continued until the specimen failed. The Lehr Fatigue Test method, regarded as an accelerated testing approach, seeks to estimate the fatigue life of materials in a significantly reduced timeframe while ensuring the collection of reliable data. This method is particularly advantageous for testing composite materials and structural components, where traditional fatigue testing methods can be prohibitively expensive and time-consuming. A notable feature of the Lehr method is its adaptability to various material types, especially in the context of biodegradable composites and fiber-reinforced polymers [39]. During the testing process, parameters such as energy dissipation, deformation, and surface temperatures were systematically monitored [38]. The Lehr method is characterized by its adaptability in experimental design, which facilitates targeted investigations into the fatigue behavior of materials. The analysis of results involves recording the stress level and the number of cycles for each stage, followed by determining the failure threshold based on the stress level at which the sample fails. Furthermore, data extrapolation can be employed to ascertain the fatigue limit for extended cycles. The Lehr method offers several advantages: it enables a more rapid determination of the fatigue limit compared to traditional “run-out” tests, which continue until a fixed number of cycles without sample failure; it is effective for materials exhibiting high variability in properties, such as polymer composites, and it conserves time and resources in industrial research. However, the Lehr method also presents certain disadvantages: the results may be influenced by the magnitude of the load steps, with excessively large increments potentially leading to erroneous outcomes, and the method presupposes the absence of hidden defects in the material that could affect the results. The Lehr method is particularly effective for applications involving structural elements in sectors such as aviation and automotives [40].

## 3. Results and Discussion

### 3.1. The Influence of Microsilica on the Water Absorption and Diffusion Coefficient of Biopolyamides

**Figure 1:**
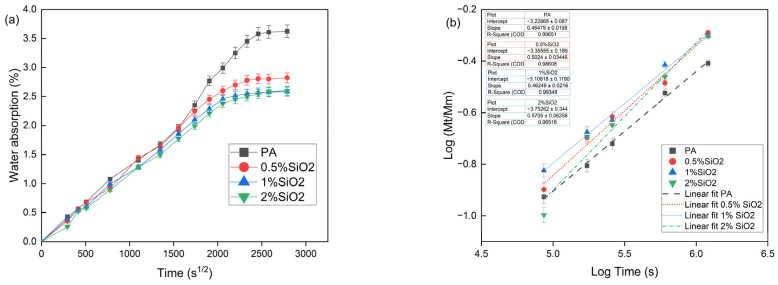
(**a**) water absorption and (**b**) comparative analysis of diffusion rates in manufactured materials.

In the context of plastics, water absorption is a significant factor. Prolonged contact between water and polymers allows water molecules to diffuse into the material. The presence of microcracks within the polymer can further facilitate the penetration of water molecules due to capillary forces. Water within the polymer structure induces both physical and chemical changes, such as hydration, which involves the binding of macromolecules to water; the formation and stabilization of ordered molecular structures; and the formation and disruption of hydrogen bonds [41,42]. The initial phase of this study involved examining the impact of microsilica on the water absorption properties of biopolyamide. Three composite variants were manufactured, each containing varying microsilica concentrations ranging from 0.5 to 2 wt.%. The incorporation of increased amounts of microsilica resulted in processing challenges and heightened material brittleness. Table 3 presents data on density, impact strength, and the diffusion coefficient along with its kinetics. An increase in the additive content led to a slight rise in material density, while impact test results did not reveal significant alterations in resistance to dynamic impact with varying modifier content. It is noteworthy, however, that the introduction of microsilica inherently diminishes the material’s capacity to absorb dynamic energy, thereby increasing stiffness and brittleness compared to the base material. This study’s findings presented in Figure 1a indicate that incorporating microsilica results in a reduction in water absorption as the additive content increases. Specifically, the water absorbency of pure polyamide, after 90 days of immersion in water, was measured at 3.6%. The introduction of microsilica at a concentration of 0.5 wt.% decreased the water absorption to 2.8%. Further increases in microsilica content resulted in water absorption levels of 2.6% for composites containing 1 and 2 wt.% microsilica. Increasing microsilica content in the polyamide composite from 1% to 2% by weight does not result in a further reduction in water absorption, which may initially appear counterintuitive given the expectation that additional barrier additives would enhance moisture protection. This phenomenon can be comprehensively explained by considering the physicochemical mechanisms of water diffusion in polymers and the inherent properties of microsilica, as corroborated by both our research findings and the existing literature. At lower concentrations, microsilica effectively occupies micropores and voids within the polymer matrix. Its fine-grained structure and chemical inertness restrict the penetration of water molecules into the composite’s interior. However, beyond this threshold, the barrier effect reaches saturation; adding an additional 1% does not significantly reduce absorbency further, as an available space within the material’s microstructure is largely occupied, and additional particles do not enhance structure as effectively [43,44]. Furthermore, increased microsilica content is linked to the formation of agglomerates, a phenomenon frequently cited in the literature as limiting the efficacy of nanometric and micrometric additives. These particles, rather than dispersing uniformly within the matrix, form clusters that locally disrupt the structural continuity, potentially leading to stress formation and microcracks [45]. Such microdefects serve as points that facilitate water penetration via a capillary mechanism, potentially negating the previously achieved protective effect. This phenomenon is further exacerbated by an increased brittleness of the material observed at higher microsilica concentrations. Our studies indicate that an increased stiffness of the material at 2% microsilica correlates with a reduced capacity to absorb dynamic energy, resulting in diminished resistance to microcracking. Consequently, instead of further reducing absorbency, microcracks form, which can act as diffusion pathways for water molecules [46]. It is also important to note that processing composites with high microsilica content becomes more technologically challenging. An increase in system viscosity and differences in flow characteristics between composite phases can lead to microstructural inhomogeneity, further degrading barrier properties. These findings align with observations from other researchers who have examined the impact of inorganic additives on water diffusion in polymeric materials [47,48]. In conclusion, increasing the microsilica content in a composite from 1% to 2% does not result in a further decrease in water absorption, likely due to one or more factors, such as saturation of the barrier effect, potential particle agglomeration, increased material fragility, and processing limitations. Further increasing the additive amount without enhancing its dispersion and microstructure control may even compromise the material’s moisture resistance properties.

Water ingress into composite materials primarily occurs through diffusion, a mechanism characterized by the direct diffusion of water molecules into a matrix and, to a lesser extent, into fibers. This process is influenced by factors such as the angle between the direction of penetration and the fibers. Additional mechanisms for moisture ingress include capillarity and transport through microcracks, which are activated only after specific damage to composites has occurred. Such damage, which facilitates increased moisture penetration by activating these additional mechanisms, often results directly from the composite’s exposure to moisture. The capillary mechanism involves a movement of water molecules along the fiber–matrix interface, followed by diffusion from this boundary into a polymer mass. This mechanism is not active unless fiber–matrix separation has occurred, typically due to water-induced degradation at the interface. Moisture transport through microcracks involves both a flow and storage of water within microcracks or other forms of micro-damage resulting from environmental influences [41]. The research conducted on water absorption facilitated the determination of the rate of water diffusion in the manufactured materials. The results of these calculations are presented in Table 3 and Figure 1b. The parameter n ranges from 0.46 to 0.57. The base material and composites containing up to 1 wt.% of microsilica exhibit Fickian diffusion, which describes the standard diffusion process in accordance with Fick’s law. In this process, molecules move uniformly and freely along the concentration gradient. The material containing 2 wt.% microsilica is characterized by a value of n=0.57, indicating an intermediate process between classical and non-Fickian diffusion, where diffusion is either accelerated or decelerated depending on the specific characteristics of the system [49]. The chemical composition of the polymer matrix has a significant influence on the solubility and diffusion of small molecules. Polymers containing polar groups, such as polyacetals or nylons, tend to attract polar molecules, such as water, rendering them hygroscopic. In polar matrices, an increase in molecular concentration can induce structural changes, including swelling or cracking. The free volume, which arises from the interstitial spaces between polymer chains, impacts the rate of absorption and diffusion, with a larger free volume facilitating greater molecular mobility. This effect is modulated by the polymer’s crystallinity, the processing method employed, and the presence of voids. Additives, such as fibers and particles, alter these properties by either enhancing or reducing absorption, contingent upon their solubility. Furthermore, interfacial boundaries can either accelerate or decelerate diffusion, depending on their interaction with the particles [50,51]. The produced materials were also subjected to static tensile tests. The test results showed that the introduction of microsilica causes a slight increase in the tensile strength and elasticity modulus. The test results showed that mechanical properties increase with the increase in microsilica content. In connection with the above, a microsilica content of 2% by weight was selected for further tests.

### 3.2. Hybrid Composites Modified with Microsilica and Short Aramid, Basalt, and Carbon Fibers

#### 3.2.1. Water Absorption of Hybrid Composites

The subsequent phase of the research involved analyzing the impact of microsilica on the properties of hybrid composites reinforced with aramid, basalt, and carbon fibers. The fiber content in the composites was maintained at 5% and 10% by weight, while the microsilica content was consistently 2 wt.%. The primary parameters influencing the mechanism of moisture sorption are the chemical composition and microstructure of the polymers. Moisture diffusion in polymer composites is governed by three distinct mechanisms already mentioned. The first mechanism involves the diffusion of water molecules through micro-gaps between polymer chains. The second mechanism involves the transfer of moisture through gaps and cavities at the fiber–matrix interface, resulting from inadequate wetting and impregnation during the initial production process. The third mechanism involves the transport of water molecules through microcracks in the matrix, which originate from the production process. Cracks in the composite, caused by the increased dimensions of the fillers, enhance the transport of moisture within the solid. Samples with a higher fiber content generally exhibit increased water absorption. Furthermore, the capillary mechanism is frequently activated, allowing water molecules to traverse the fiber–matrix boundary, resulting in increased diffusivity [49].

**Figure 2:**
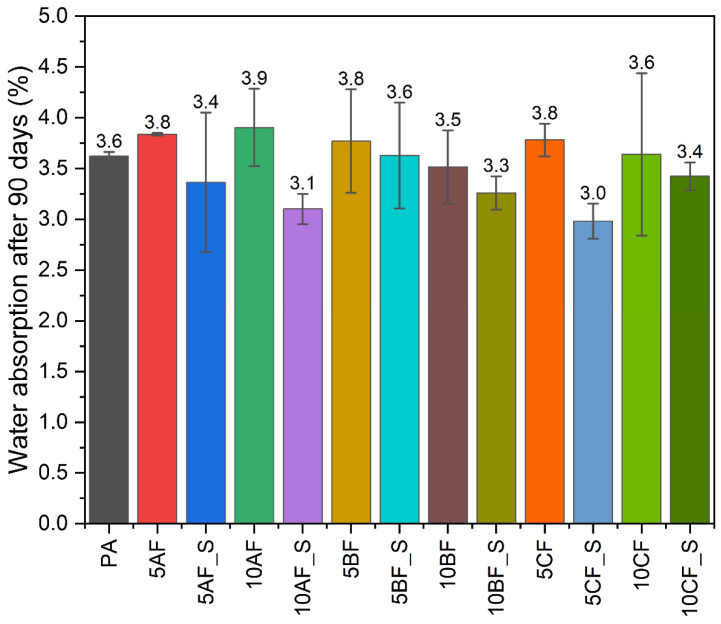
Results of water absorbency following a 90-day incubation period of samples in water.

Figure 2 illustrates the water absorption characteristics of the fabricated materials following a 90-day incubation period in water. The findings indicate that incorporating fibers into polyamide results in a modest increase in water absorption, approximately 5%, compared to the base material. For other composites, the water absorption levels without the inclusion of microsilica remain comparable to those of pure polyamide. Notably, the addition of 2 wt.% microsilica significantly reduces water absorption by approximately 20% in carbon fiber composites and silica, relative to pure polyamide.

Analysis of the diffusion coefficient indicates that water disperses within the composites at a comparable rate (Table 4). The constant rate, denoted as k, varies among different composites and is influenced by material properties such as porosity and reinforcement geometry. Although the variations in the k parameter are minor, it can be inferred that incorporating microsilica into the material reduces the rate of water transport into the material’s interior, likely by filling existing porosities. An increase in the k constant signifies an enhanced rate of liquid transport within the polymer. Porous materials or those with more defects, such as microcracks, exhibit a more rapid flow of substances. In polymers and polymer composites, this can lead to accelerated degradation [52]. Analysis of the parameter n revealed that nearly all the materials tested exhibited abnormal diffusion.

Aramid fibers are characterized by their low moisture absorption and robust interfacial bonding, attributed to their crystalline structure and polar chemical composition. Their incorporation led to a moderate increase in water absorption due to fiber–matrix interfacial effects yet overall contributed to maintaining composite integrity. The capillary effect at the fiber–matrix interface becomes more pronounced with increased fiber content (5–10 wt.%), slightly enhancing diffusion and water absorption. Basalt fibers, being mineral-based and less hydrophilic than aramid or natural fibers, demonstrated strong resistance to water absorption. However, at higher fiber content, they facilitated the formation of microcracks due to a mismatch between stiffness and the polymer, which increased capillary water ingress through interfacial pathways. Despite this, the integration with microsilica significantly reduced water penetration by pore filling and surface sealing. Carbon fibers exhibited the most substantial effect on reducing water absorption following microsilica modification. Their inherent low hygroscopicity and the silica barrier effect resulted in the lowest water absorption among the tested composites (reduced by ~20% compared to unmodified composites). The diffusion coefficient was also lowest in the carbon fiber-reinforced composites, indicating effective inhibition of moisture transport. In summary, the specific fiber types—aramid, basalt, and carbon—exhibit distinct behaviors concerning water absorption. These differences are attributable to their hydrophilicity, geometry, surface energy, and the nature of their interfacial bond to the biopolyamide matrix. The presence of microsilica further modifies these effects, reducing porosity and obstructing capillary pathways.

Understanding diffusion processes is crucial due to their impact on several fundamental properties of polymer materials. Composites characterized by a high diffusion coefficient may exhibit reduced efficacy as barriers to gases and liquids, a factor of particular significance in packaging applications designed to protect against moisture or oxygen ingress. Conversely, a lower diffusion coefficient enhances the material’s resistance to the penetration of aggressive chemicals, which is vital in challenging environments. Furthermore, diffusion influences the interactions between the polymer matrix and fillers, thereby affecting the composite’s mechanical properties, including its strength and elasticity [53]. In composites incorporating mineral reinforcing fillers, the influence of the aquatic environment manifests differently compared to composites with natural fibers. Empirical research has substantiated the hydrophobic properties of both fibers and mineral particles, which are characterized by minimal water absorption. The polymer’s propensity to absorb moisture diminishes as the fiber content increases, attributable to the reduction in the hygroscopic phase within the matrix. Nevertheless, the specific type of fiber can modify water absorption due to capillarity effects, thereby influencing both the water content and the absorption rate. Notably, moisture penetration in polymer composites occurs 100 to 400 times more rapidly along the fiber than through the matrix [54]. The incorporation of coupling agents can mitigate water diffusion by decreasing the formation of capillaries at the interface between the fiber and the matrix. Additionally, the presence of voids, which may arise from trapped air during the manufacturing process or from vaporized components during the high-temperature curing cycle of composites, can enhance the diffusivity of moisture into the composites [55]. In composites reinforced with mineral fillers, water absorption can lead to polymer swelling, thereby inducing tensile stress at the fiber–matrix interface. This occurs because the fibers’ stiffness restricts the polymer’s expansion. Additional effects of water absorption include the plasticization of the polymer, resulting from the displacement of polymer molecules, and the weakening of the interphase between the fiber and the matrix. While the detachment of the fiber from the matrix constitutes a permanently destructive mechanism, the plasticizing effect of water is reversible upon the desorption of water [56,57]. Ishak and Lim [58] conducted an investigation into the impact of moisture absorption on the tensile properties of short glass fiber-reinforced polybutylene terephthalate (PBT). It was observed that the equilibrium moisture content increased as the fiber content decreased, attributable to the increased volume fraction of the hygroscopic phase, namely the matrix. Specifically, the equilibrium moisture content for unreinforced PBT and PBT reinforced with a 17% fiber volume fraction was determined to be 0.85% and 0.7% by weight, respectively. A slight reduction in diffusivity was noted in fiber-modified materials compared to the pure polymer, which was attributed to the distribution of fibers that impede direct moisture diffusion. A significant reduction in tensile strength and the modulus of elasticity was observed upon exposure to moisture. The effects of moisture were more pronounced at 100% relative humidity compared to 81% relative humidity, due to the intensified plasticization and hydrolysis of the matrix, as well as the degradation of the fiber–matrix interface. Alexis et al. [59] conducted an investigation into the impact of moisture absorption on the mechanical properties of glass fiber-reinforced polyamide. Tensile tests were performed on both dry and saturated samples at relative humidity levels of 50% and 100%. The results indicated a decrease in tensile strength and elastic modulus with increasing humidity, while an increase in strain at break was observed. Under wet conditions, matrix plasticization and interfacial delamination between the fiber and the matrix were predominant, whereas in the dry state, brittle cracking of the matrix occurred without evidence of matrix deformation. An alternative approach to mitigating moisture absorption involves the hybridization of fibers with more hydrophobic inorganic fillers and/or synthetic fibers. Empirical studies have demonstrated that the incorporation of fibers and inorganic particles can effectively reduce moisture absorption in natural fiber-based composites. Specifically, the addition of fly ash, heavy calcium carbonate, and SiO_2_ resulted in reductions in water absorption by 1.8%, 25.8%, and 42.4%, respectively, when compared to composites composed solely of natural fibers. Furthermore, the inclusion of SiO_2_ was particularly effective in diminishing water absorption as its concentration increased. SiO_2_ functions as a barrier agent, impeding the ingress of water into the composites, thereby reducing both water absorption and the diffusion coefficient. Similar findings were observed in natural fiber composites reinforced with aluminum hydroxide (Al(OH)_3_), magnetite, and magnesium carbonate (MgCO_3_). The integration of these inorganic fillers significantly decreased the moisture absorption and swelling of the hybrid composites. The substantial enhancement in the waterproofing properties of the composites can be attributed to the adhesion between the fibers and inorganic fillers, which is facilitated by the formation of hydrogen bonds. This interaction effectively blocks a significant number of –OH groups that could otherwise interact with water molecules [60].

#### 3.2.2. Basic Mechanical Properties

**Figure 3:**
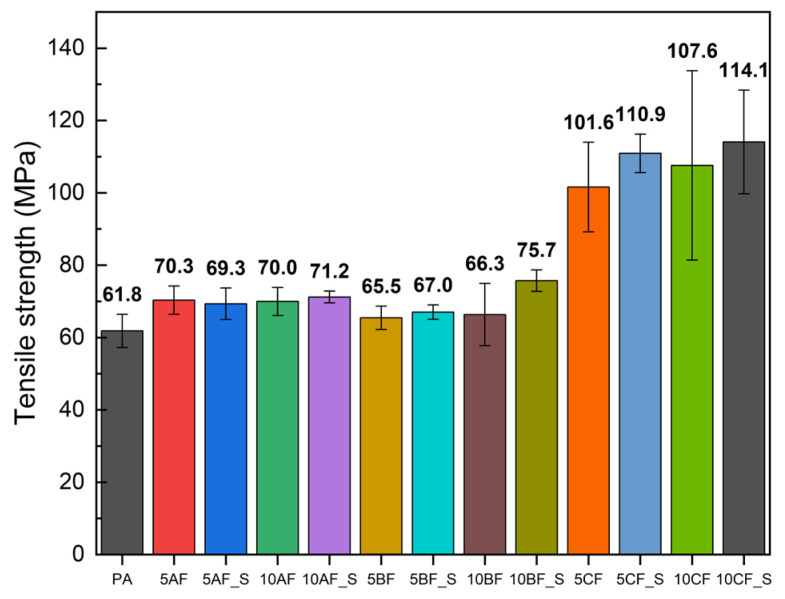
Comparison of tensile strength of manufactured composites.

**Figure 4:**
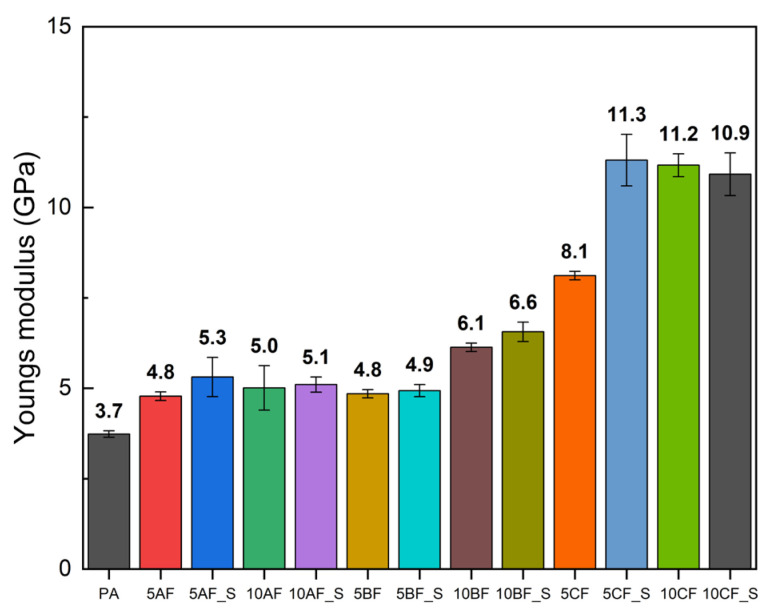
Comparison of Young’s modulus of manufactured composites.

**Figure 5:**
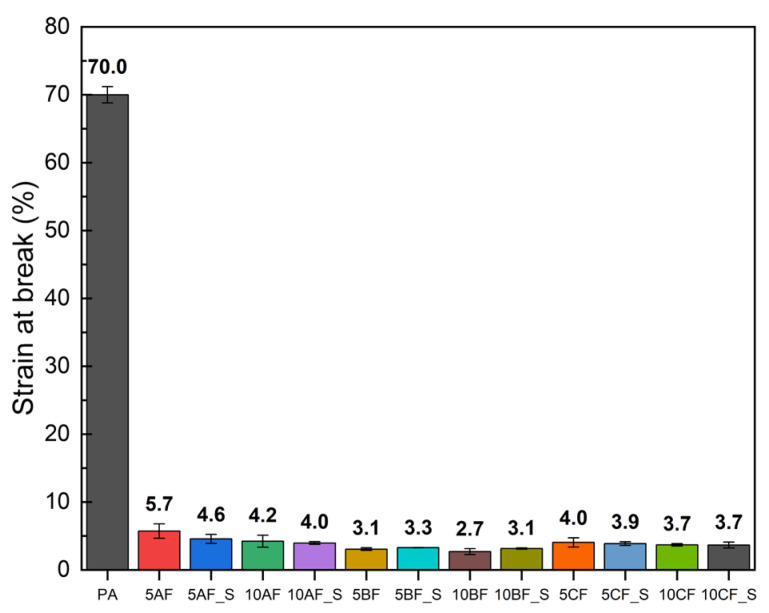
Comparison of strain at break of manufactured composites.

The manufactured materials underwent mechanical evaluation through static tensile testing, with the findings depicted in Figure 3, Figure 4 and Figure 5. This study’s results indicate that the incorporation of silica microparticles into the composites results in a marginal increase in tensile strength and a somewhat more pronounced enhancement in the elastic modulus. The most significant effect was observed in composites containing basalt fiber, which may be attributed to the similar chemical composition of basalt fibers and microsilica [61]. Composites reinforced with carbon and basalt fibers exhibited significant variability in results, highlighting the challenges associated with achieving a uniform composite structure during manufacturing. The incorporation of fiber and particle reinforcement markedly diminishes the deformation at break. The integration of reinforcing fibers into polymer composites can substantially influence deformation at break, a critical parameter of the material’s mechanical properties. The subsequent discussions elucidate key factors contributing to this alteration in strain at break. The primary cause for the reduction in strain at break is the increased stiffness imparted by the reinforcing fibers. Mansor et al. demonstrated that kenaf fibers, when incorporated into thermoplastic matrices such as polypropylene and polyamide 6, enhance the overall stiffness of the composite material due to their crystalline structure and high cellulose content [62]. This augmented stiffness can enhance tensile strength but often results in a concomitant reduction in strain at break, as the material becomes less prone to bending and elongation prior to failure. Additionally, fiber–matrix interaction plays a pivotal role in determining mechanical properties. When the adhesion between the fiber and the polymer matrix is inadequate, stresses are concentrated at the fiber–matrix interface, as noted by Etcheverry and Barbosa [63]. Insufficient adhesion can lead to damage or fiber pull-out, causing the material to exhibit brittle behavior and resulting in reduced deformation at break. A robust interfacial bond is essential for effective load transfer, whereas a weak bond compromises the overall ductility of the composite. Furthermore, the geometry of the reinforcing fibers significantly influences the deformation at break. Xu et al. discuss how fiber length and volume fraction can modify the mechanical properties of a composite. Shorter fibers can promote increased void formation in the matrix by acting as stress concentrators, leading to premature failure under tensile loading [64]. This suggests that optimal fiber length and distribution are necessary to achieve a balance between stiffness and ductility, with poorly graded composites exhibiting diminished strain at break. Moreover, the type of reinforcing fiber can affect the deformation at break. Won et al. highlighted that polyarylate (PAR) and nylon 6 composites exhibit distinct mechanical behaviors; PAR fibers can enhance tensile strength but may decrease elasticity, particularly under dynamic loading conditions [65]. While stronger fibers can increase load-bearing capacity, they may inadvertently lead to a reduction in elongation before failure. Another significant factor is the concept of fiber hybridization, as investigated by Saba et al. They found that combining natural fibers with nanofillers can enhance mechanical properties, including tensile strength, while causing some deterioration in strain at break due to the stiffness imparted by nanofillers [66]. Thus, while hybrid composites can offer improved performance, careful management of component ratios is necessary to avoid excessive rigidity.

The interaction between reinforcing fibers and microsilica powder in the context of injection molding presents several challenges that impact both the processing and the properties of composite materials. The incorporation of microsilica can enhance the mechanical properties of fiber-reinforced composites, including tensile and flexural strength, due to its fine molecular structure and matrix compaction capability [67]. Nonetheless, the injection molding process, when conducted without prior compounding reinforcement with the matrix, can lead to the formation of agglomerates, which subsequently reduces strength properties. A primary challenge in the injection molding of staple fiber and microsilica composites is the presence of shear forces. As demonstrated by Hwang et al., these forces can cause fiber shortening, thereby reducing their aspect ratio and diminishing the reinforcement’s effectiveness [68]. The reduction in fiber length adversely impacts the mechanical properties of the molded component. Furthermore, the presence of microsilica may intensify fiber degradation due to heightened friction and wear within injection devices, thereby compromising their structural integrity [67].

Variations in the flow characteristics of fibers and microsilica pose a significant challenge in achieving a homogeneous composite matrix. It is essential to ensure the uniform dispersion of fibers, as their agglomeration can result in zones of structural weakness within the composite [69]. Microsilica, in response to the stresses generated during the injection process, can lead to inconsistent packing and compromise the stress transfer between the fiber and the matrix [70]. Furthermore, the fine particle size can result in complex flow dynamics and increase the viscosity of the molten material, thereby impeding processing [71,72]. While microsilica enhances certain mechanical properties, the fiber content may be insufficient to fully and effectively reinforce the composite.

The molding temperature is also a critical parameter influencing the interaction between microsilica and reinforcing fibers. Jeon et al. observed that inappropriate temperature profiles can result in incorrect matrix melting and inadequate integration of fillers such as microsilica, thereby impairing fiber adhesion [73]. In summary, while the incorporation of microsilica into chopped fiber composites can enhance their mechanical properties, it necessitates addressing several processing challenges. Critical considerations include preserving fiber integrity under shear forces, ensuring uniform dispersion without agglomeration, managing viscosity at high fiber content, and optimizing processing temperatures for each type of composite separately, which can significantly influence the structural and thus mechanical properties of the composite, improving their mechanical properties. Each of these factors can influence the properties of the final composite and its performance in practical applications.

Static bending tests were conducted on composites and hybrid composites containing 5% fiber content, with the results detailed in Table 5. These tests were performed on both conditioned samples and samples subjected to 90 days of water incubation. The findings indicate that the incorporation of microsilica into the composites results in an enhancement of both the flexural strength and flexural modulus. The observed increase in flexural strength following the application of microsilica is likely due to the particle blocking effect, which frequently impedes the movement of polymer segments [74]. The research conducted following the incubation of samples in water elucidates the plasticizing effect of water on the compositions under study. All composites exhibited a significant reduction in bending properties, characterized by a decrease in bending strength exceeding 60% and an almost twofold reduction in the modulus during bending.

In the context of plastics and composites, significant alterations in their matrix are primarily induced by aging processes, which critically determine the material’s suitability for specific environmental applications and its overall lifespan. Polymer aging refers to the degradation of polymers due to the cumulative impact of physical and chemical factors encountered during usage and storage. In linear polymer materials, degradation results in the shortening of macromolecular chains, leading to a reduction in molar mass. For polymers with more complex chain structures, in addition to the cleavage of the main polymer chain, reactions involving the fracture of side groups also occur. Physical factors such as temperature and moisture contribute to destructive changes in the chemical and physical structure of polymers, often resulting in alterations and typically a reduction in their strength properties [75].

The water resistance of composites is contingent upon the polymer constituting the matrix of the composite, the filler, and the manner in which the matrix is bonded to the filler. Within the composite–water system, both chemical and physical alterations, as previously mentioned, may occur. Additional changes induced by water include the swelling of the material, resulting from the migration of water molecules into the interior of the material. This process generates tensile stresses at the swelling front, which may lead to the formation of microcracks and a reduction in mechanical properties [76,77]. The research findings demonstrate that while the incorporation of microsilica effectively reduces water absorption, it does not mitigate the influence of the aquatic environment on the properties of the manufactured composites.

#### 3.2.3. Microstructure of Manufactured Composites

**Figure 6:**
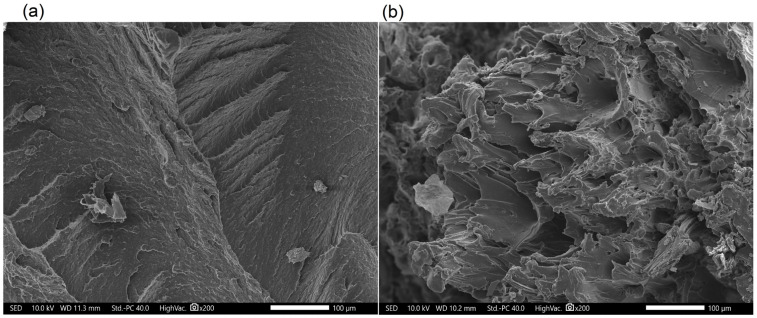
Microstructure of polyamide: (**a**) neat biopolyamide; (**b**) biopolyamide modified 2 wt.% of microsilica.

**Figure 7:**
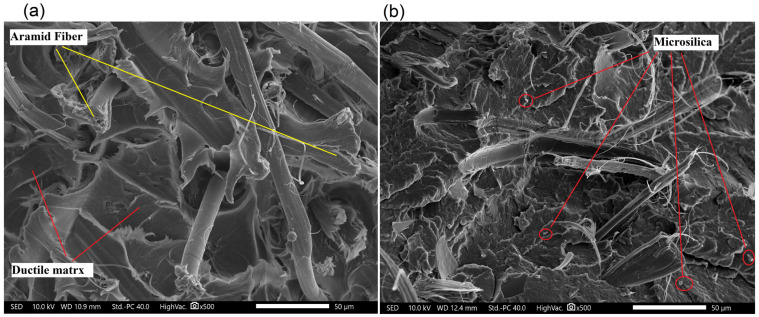
Microstructure of polyamide composites: (**a**) 5AF; (**b**) 5AF_S.

**Figure 8:**
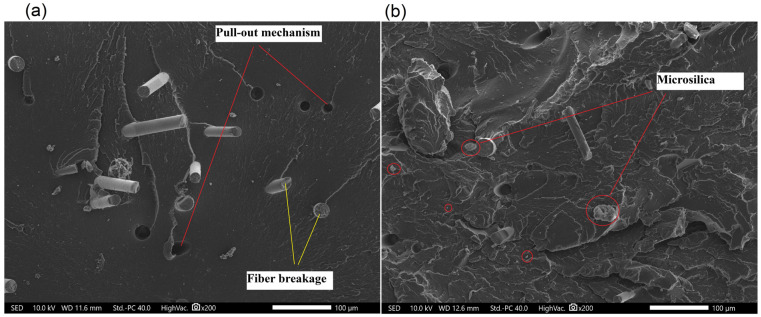
Microstructure of polyamide composites: (**a**) 5BF; (**b**) 5BF_S.

**Figure 9:**
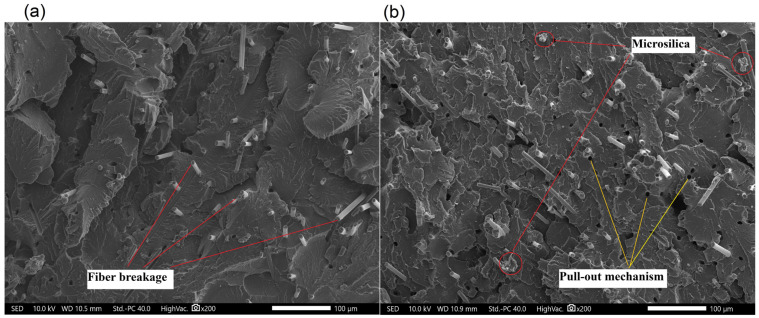
Microstructure of polyamide composites: (**a**) 5CF; (**b**) 5CF_S.

During the research, microscopic examinations of the manufactured materials were conducted. The microstructures are depicted in Figure 6, Figure 7, Figure 8 and Figure 9. A significant influence of the fibers on the alteration of the fracture characteristics of the polymer matrix was observed. Polyamide, as a base material, is characterized by a highly ductile fracture, as corroborated by the results obtained from the static tensile test, where the deformation at break of polyamide exceeded 70%. The incorporation of aramid fibers does not induce substantial changes in the polymer structure, and the fracture remained ductile. Microscopic images reveal the developed nature of the matrix and the aramid fibers, which were well integrated into the composite matrix. More pronounced structural changes were observed in composites with basalt and carbon fibers. Basalt fiber exhibits a smooth character, while the matrix displays a brittle nature and does not envelop the fiber, indicating relatively poor adhesion of basalt fibers to polyamide. Two cracking mechanisms were identified, namely fiber breakage and fiber pull-out from the matrix, with neither being the predominant mechanism. The microstructure of carbon fiber composites also demonstrates brittle fracture; however, in this instance, the dominant mechanism was fiber fracture, suggesting better adhesion of carbon fibers to the polymer matrix, which resulted in enhanced strength properties. Only a few microsilica particles were observed in the microscopic images. The tensile test results indicate that the incorporation of microsilica leads to a modest enhancement in tensile strength and a more significant increase in the modulus of elasticity. However, microstructural analysis reveals the presence of only a limited number of microsilica particles, suggesting their uneven distribution within the matrix, which may constrain their full reinforcing potential. Concurrently, microsilica influences the structural density, accounting for the observed increase in stiffness, despite the absence of a discernible effect on microstructural homogeneity. In mechanical testing, composites reinforced with basalt fibers exhibited the most substantial improvement in mechanical properties with the inclusion of microsilica, potentially attributable to the chemical composition similarity between basalt and microsilica. Nevertheless, microscopy reveals inadequate encapsulation of basalt fibers by the matrix, with cracks propagating through both tearing and fiber pull-out. This observation suggests suboptimal adhesion between the basalt fibers and the matrix, potentially compromising long-term durability despite the enhancement in stiffness. Carbon fiber composites also demonstrated fracture brittleness; however, in this instance, the predominant mechanism was fiber fragmentation, indicating superior adhesion to the matrix. This is reflected in enhanced mechanical properties, affirming that effective stress transfer is contingent upon the quality of the fiber–matrix bond rather than solely on the presence of microsilica. The challenges encountered in injection molding, such as microsilica aggregation, fiber shortening due to shear forces, and increased system viscosity, adversely affect microstructural homogeneity and diminish the reinforcing effect. These issues align with micro-observations indicating irregular microsilica distribution and limited fiber–matrix bonding. To summarize:

- The effectiveness of microsilica reinforcement depends on uniform dispersion and processing quality—without prior compounding, agglomerates appear, which weaken mechanical properties;
- The best adhesion and effective load transfer were observed in composites with carbon fibers, which correlates with their microstructure and the dominance of the fiber fracture mechanism;
- Basalt fibers combined with microsilica improved mechanical properties, but their poor adhesion in the microstructure may limit long-term durability.

#### 3.2.4. Accelerated Fatigue Testing

In the study of accelerated fatigue parameters, an initial load equivalent to 5% of the material’s tensile strength was applied, with the amplitude subsequently increased by an additional 5% after every 1000 cycles. The experiment continued until the sample exhibited damage. Mechanical hysteresis loops, maximum elongation, and maximum dissipation energy were recorded at each load level. Based on these data, the failure threshold was determined using the Lehr method as a comparative parameter for composites tested under identical conditions. The failure threshold ($\sigma_{x}$) in the Lehr method represents a theoretical stress level that delineates the boundary between safe and hazardous fatigue load ranges. This threshold is the value below which the material is theoretically not expected to sustain damage during prolonged load cycles, whereas exceeding this value results in property degradation and eventual failure. Fatigue strength is defined as the maximum stress a material can endure for an indefinite number of cycles without the risk of failure. This parameter is more precisely determined through long-term fatigue tests. For materials such as steel, fatigue strength is characterized by a well-defined value below which failure does not occur. In contrast, for materials such as aluminum or polymer composites, fatigue strength is determined by the number of cycles, for example, 10^6^ or 10^7^ [78].

The failure threshold serves as a more rapid indicator for evaluating the onset of material degradation in terms of durability; however, it lacks the precision of a classical fatigue method. In the Lehr method, the failure threshold is established based on two distinct stress levels:

(6) $$\sigma_{f}={2\sigma }_{n-1}-\sigma_{n},$$

where

$\sigma_{n-1}$—the stress at which the material has withstood a full load cycle without being destroyed, (MPa);
$\sigma_{n}$—stress at which the material was destroyed, (MPa).

**Figure 10:**
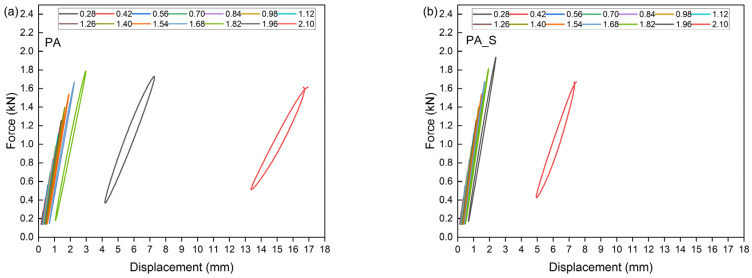
Hysteresis loops for biopolyamide (**a**) and composite containing 2 wt.% of microsilica (**b**).

**Figure 11:**
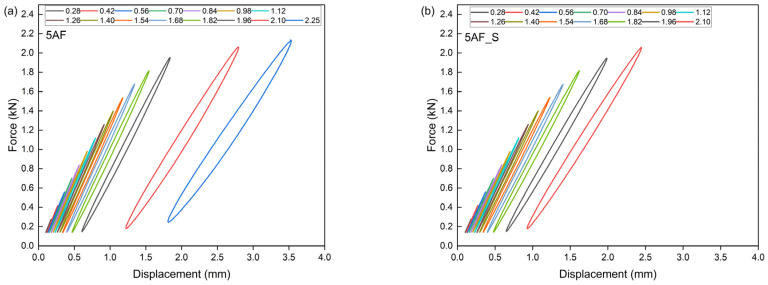
Hysteresis loops for composites containing 5 wt.% of aramide fibers (**a**), as well as for hybrid composites further modified with an additional 2 wt.% of microsilica (**b**).

**Figure 12:**
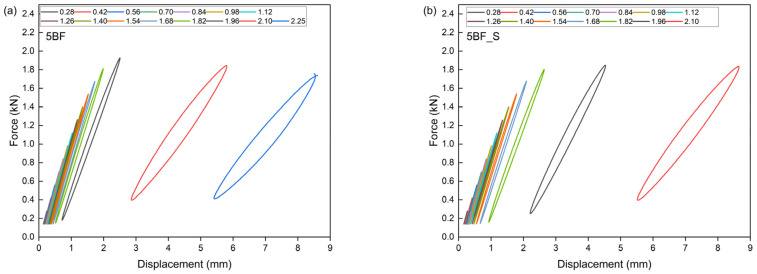
Hysteresis loops for composites containing 5 wt.% of basalt fibers (**a**), as well as for hybrid composites further modified with an additional 2 wt.% of microsilica (**b**).

**Figure 13:**
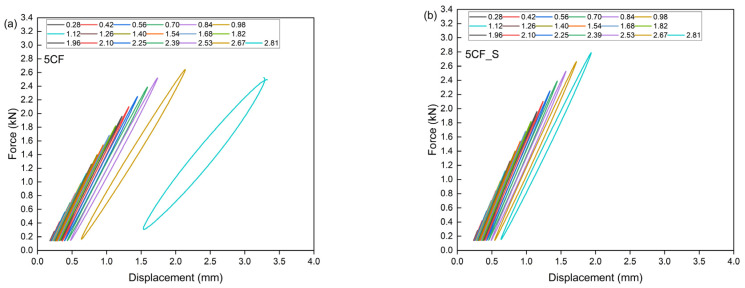
Hysteresis loops for composites containing 5 wt.% of carbon fibers (**a**), as well as for hybrid composites further modified with an additional 2 wt.% of microsilica (**b**).

**Figure 14:**
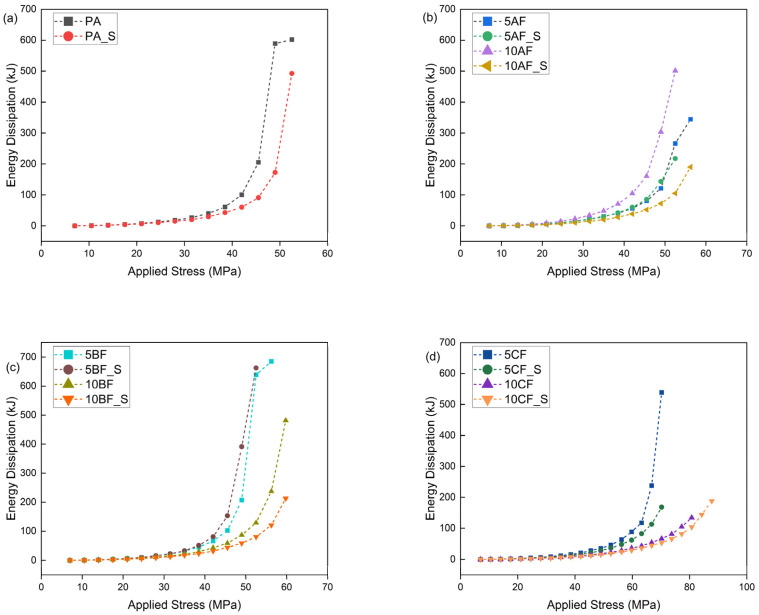
Dependence of dissipation energy on applied stress: (**a**) polyamide and composites with microsilica, (**b**) biopolyamide composites and hybrid composites with aramid fiber, (**c**) biopolyamide composites and hybrid composites with basalt fiber, and (**d**) biopolyamide composites and hybrid composites with carbon fiber.

This theoretical value signifies the threshold at which a material becomes unstable under cyclic loading conditions. The Lehr method facilitates the estimation of the material’s approximate fatigue strength. This method is predicated on the theory that, prior to crack initiation under cyclically variable loads, there is a significant increase in energy dissipation. This increase in energy is attributed to the mechanisms occurring within polymer composites, which involve the conversion of energy into thermal energy and friction phenomena between composite components, as well as the movement of macromolecules, the separation of reinforcement from the matrix, and the extraction of fibers from the material’s matrix [38,79]. Table 6 presents the parameters determined by the accelerated fatigue method, including $\sigma_{n-1}$—stress level without failure; $\sigma_{n}$—stress level at failure; $\sigma_{f}$—failure threshold; $Z_{z}$—approximate fatigue strength; U—ultimate tensile strength. Figure 10, Figure 11, Figure 12 and Figure 13 show the translative hysteresis loops recorded during accelerated fatigue testing for composites with 5 wt.% of fibers. Figure 14 shows the value of the dissipation energy in relation to the applied stress level, on the basis of which the approximate fatigue strength was determined. The area enclosed by a hysteresis loop represents the energy dissipated during a loading cycle. This dissipated energy is the difference between the energy input during loading and the energy recovered during unloading. Energy dissipation was calculated using specialized software (Autograph Trapezium X, https://www.shimadzu.com/an/products/materials-testing/uni-ttm-software/trapezium-x/index.html). A detailed description of the method can be found in [38].

The research findings, beyond the evident conclusions, pertain to the utilization of reinforcing fibers and their beneficial impact on fatigue strength, particularly with carbon fibers. The introduction of microsilica into composites has demonstrated an approximate 15% increase in fatigue strength for the base material modified with 2 wt.% microsilica, a 5% increase for composites with aramid fiber, and a 10–15% increase for composites with basalt and carbon fiber, compared to those not modified with microsilica. Furthermore, the application of microsilica significantly reduced the dynamic creep of polyamide. It is important to note that the method presented is approximate and does not provide definitive results regarding fatigue strength; however, it serves as an effective and expedited method for comparing materials tested under identical load conditions. The addition of microsilica to polymer composites has been shown to enhance their fatigue strength and overall mechanical properties. Microsilica, also referred to as silica dust, functions as a microfiller within the composite matrix, altering mechanical properties through mechanisms such as particle dispersion and enhancement of the transition zone. Studies suggest that its presence increases compressive, tensile, and flexural strength in cementitious composites, and these findings are applicable to polymer composites due to similar bonding mechanisms. Microsilica enhances the formation of a denser microstructure by filling voids and strengthening the bond between polymer chains [75], thereby leading to an improvement in mechanical properties, including fatigue resistance. This enhancement reduces the likelihood of crack initiation and propagation under cyclic loading [47]. Research indicates that the fatigue behavior of polymer composites is significantly influenced by the extent of reinforcement and the structural orientation of the fibers within the matrix [80,81]. The incorporation of microsilica has been demonstrated to enhance not only the elastic modulus but also the material’s strength, thereby improving its capacity to absorb energy under cyclic loading conditions [82]. This consideration is crucial for applications exposed to repetitive forces, particularly within the domains of automotive and aerospace components [83]. Furthermore, the interaction of microsilica with other composite components can enhance the viscoelastic properties, which are crucial for the material’s performance under fatigue conditions. The incorporation of microsilica reduces viscoelastic deformations, thereby improving the composite’s resistance to creep and fatigue [84]. In experimental evaluations, samples incorporating an optimal concentration of microsilica demonstrated enhanced fatigue life, exhibiting superior reliability and sustained performance under cyclic loading conditions compared to samples lacking this additive [85]. Furthermore, microsilica influences the microstructural characteristics, facilitating a more uniform distribution of stress within the composite material and reducing local stress concentrations that may result in fatigue failure [86]. Contemporary multi-scale modeling methodologies have yielded enhanced insights into the influence of microsilica on fatigue properties across various loading conditions, substantiating its beneficial effect on augmenting mechanical performance [87].

Analysis of the mechanical and fatigue test results revealed a distinct correlation between the enhancement of the static properties of the composites and their resistance to cyclic loading. Notably, composites incorporating carbon fibers and microsilica modification exhibited the highest tensile strength (up to 114 MPa) and elastic modulus (>10 GPa), which directly contributed to increased fatigue strength ($Z_{z}$~56.3 MPa for 10CF_S). Conversely, materials with aramid fibers, despite improvements in Young’s modulus, demonstrated lower fatigue resistance ($Z_{z}$~31.5 MPa), indicating the limited efficacy of these fibers in transferring cyclic stresses. The inclusion of microsilica significantly enhanced fatigue parameters, particularly in composites with basalt and carbon fibers, by elevating their threshold damage level $\sigma_{f}$ and extending the fatigue life limit. This improvement can be attributed to reduced porosity, enhanced interfacial adhesion, and the sealing of composite microstructures, which diminish local stress concentrations and delay crack initiation. However, it was observed that the heterogeneous dispersion of microsilica and fibers (especially in 10AF_S samples) could result in the formation of agglomerates and structural microdefects, thereby reducing both tensile strength and deformation at break. These findings underscore the necessity to optimize the mixing and processing procedures, particularly with a high proportion of solids. In summary, the fatigue strength efficiency of biopolyamide composites is a function of the synergy between static parameters, microstructure quality, and the type and distribution of the fiber and microsilica used. The optimal balance between fatigue and mechanical resistance is exhibited by hybrid composites with carbon fiber modified with 2% microsilica. In conclusion, the incorporation of microsilica represents a viable strategy for enhancing the fatigue strength of polymer composites. Its ability to refine microstructure, improve interfacial adhesion, and mitigate degradation under mechanical stress underscores its effectiveness as an additive in the development of advanced composite materials.

## 4. Conclusions

In this study, biocomposites were developed using biopolyamide PA410, enhanced with microsilica, and reinforced with short aramid, basalt, and carbon fibers. The investigation focused on analyzing physicochemical and mechanical properties, fatigue strength, and water absorption capacity. The findings indicated that incorporating 2 wt.% of microsilica significantly enhances the strength properties and durability of the materials under variable load conditions. This improvement is attributed to microsilica’s ability to fill voids within the composite structure and enhance interfacial adhesion. Microsilica effectively reduced water absorption by up to 20% in composites with carbon fiber and positively influenced stress distribution within the material. The type of fiber used resulted in varying effects on water absorption, mechanical, and fatigue properties. Aramid fibers enhanced the modulus of elasticity and tensile strength; however, at higher concentrations, they facilitated moisture transport through the fiber–matrix interface, potentially leading to localized weakening of the composite structure. Basalt fibers increased stiffness and resistance to bending, and their synergistic effect with microsilica further strengthened the composite and improved fatigue resistance. Notably, carbon fibers yielded the most advantageous outcomes, enhancing all key mechanical parameters, including tensile strength and the Young’s modulus, while also minimizing water absorption, particularly after modification with microsilica. Accelerated fatigue tests conducted using the Lehr method demonstrated that composites with carbon fiber and microsilica achieved the highest fatigue damage thresholds ($\sigma_{f}$ up to 80.8 MPa) and approximate fatigue strength ($Z_{z}$ up to 56.3 MPa). Conversely, composites with aramid fiber exhibited moderate fatigue resistance, whereas those with basalt fiber demonstrated high fatigue stability, especially with reduced microporosity and improved compatibility with microsilica. In summary, hybrid composites containing carbon fiber and microsilica exhibited the highest efficacy in enhancing mechanical properties, moisture resistance, and fatigue life. The results validate the use of such modifications in applications demanding high durability and mechanical stability, particularly under cyclic load and humidity conditions. These composites are applicable in engineering contexts. Current research focuses on utilizing these materials as external connectors for terrace boards, which are exposed to environmental factors such as UV radiation and moisture, as well as cyclic loading. Consequently, this study may significantly influence the application of these manufactured materials.

## Figures and Tables

**Table 1:** Injection molding parameters to produce testing specimens.

Temperature (°C)							Injection Pressure (bar)	Compression Pressure (bar)	Press Time (s)
Feed Zone	Zone 1	Zone 2	Zone 3	Zone 4	Nozzle	Mold			
40	190	265	265	275	280	60	1200	600	8
Screw speed (RPM)			Back pressure (bar)			Injection speed (mm/s)		Gate design and location	
100			40			20		Edge gate	

**Table 2:** Description of manufactured materials.

Index	Description
PA	Bio-based polyamide 100%
0.5%SiO_2_	PA + 0.5 wt.% microsilica
1%SiO_2_	PA + 1 wt.% microsilica
2%SiO_2_	PA + 2 wt.% microsilica
5AF	PA + 5 wt.% aramid fiber
5AF_S	PA + 5 wt.% aramid fiber + 2 wt.% microsilica
10AF	PA + 10 wt.% aramid fiber
10AF_S	PA + 10 wt.% aramid fiber + 2 wt.% microsilica
5BF	PA + 5 wt.% basalt fiber
5BF_S	PA + 5 wt.% basalt fiber + 2 wt.% microsilica
10BF	PA + 10 wt.% basalt fiber
10BF_S	PA + 10 wt.% basalt fiber + 2 wt.% microsilica
5CF	PA + 5 wt.% carbon fiber
5CF_S	PA + 5 wt.% carbon fiber + 2 wt.% microsilica
10CF	PA + 10 wt.% carbon fiber
10CF_S	PA + 10 wt.% carbon fiber + 2 wt.% microsilica

**Table 3:** Results of density, impact strength, velocity, and diffusion kinetics analysis of composites.

Index	Density (g/cm^3^)	Impact Strength (kJ/m^2^)	D	Parameter k (s)	Parameter n
PA	1.08 ± 0.01	-	3.58 × 10^−13^	0.00122	0.4648
0.5%SiO_2_	1.09 ± 0.01	24.7 ± 1.8	7.24 × 10^−13^	0.00135	0.5024
1%SiO_2_	1.10 ± 0.01	22.7 ± 1.1	5.88 × 10^−13^	0.00111	0.4625
2%SiO_2_	1.14 ± 0.01	23.0 ± 1.0	7.63 × 10^−13^	0.00127	0.5705

**Table 4:** Results of density, impact strength, velocity, and diffusion kinetics analysis of composites and hybrid composites.

Index	Density (g/cm^3^)	Impact Strength (kJ/m)	D	Parameter k(s)	Parameter n
PA	1.08 ± 0.01	-	3.58 × 10^−13^	0.001220	0.4648
5AF	1.12 ± 0.01	25.6 ± 1.8	4.22 × 10^−13^	0.001403	0.5169
5AF_S	1.14 ± 0.01	28.5 ± 0.2	5.03 × 10^−13^	0.001343	0.4958
10AF	1.14 ± 0.01	23.3 ± 2.4	4.52 × 10^−13^	0.001426	0.5710
10AF_S	1.15 ± 0.01	19.2 ± 0.9	4.03 × 10^−13^	0.001296	0.5188
5BF	1.13 ± 0.01	36.0 ± 6.2	3.56 × 10^−13^	0.001270	0.5446
5BF_S	1.16 ± 0.01	31.4 ± 1.0	6.09 × 10^−13^	0.001313	0.6888
10BF	1.13 ± 0.01	33.6 ± 2.4	5.17 × 10^−13^	0.001584	0.7208
10BF_S	1.17 ± 0.01	32.2 ± 2.8	6.18 × 10^−13^	0.001375	1.0140
5CF	1.23 ± 0.04	17.9 ± 1.0	4.09 × 10^−13^	0.001267	0.4919
5CF_S	1.25 ± 0.04	16.3 ± 2.1	4.42 × 10^−13^	0.001221	0.5082
10CF	1.41 ± 0.03	36.2 ± 1.0	6.77 × 10^−13^	0.001689	0.8122
10CF_S	1.42 ± 0.01	34.5 ± 3.1	5.64 × 10^−13^	0.001451	0.7303

**Table 5:** Comparison of flexural properties before and after the water absorption process.

State	Conditioned		After Water Absorption	
Material	Flexural Strength (MPa)	Flexural Modulus (GPa)	Flexural Strength (MPa)	Flexural Modulus (GPa)
PA	121.4 ± 2.2	7.8 ± 0.1	41.2 ± 0.6	0.98 ± 0.02
PA_S	138.7 ± 4.8	8.5 ± 0.1	40.8 ± 0.5	1.01 ± 0.01
5AF	129.2 ± 0.8	8.3 ± 0.1	46.3 ± 0.9	1.24 ± 0.05
5AF_S	135.9 ± 1.1	8.7 ± 0.5	48.9 ± 2.0	1.26 ± 0.06
5BF	135.1 ± 2.4	9.0 ± 0.3	49.4 ± 3.2	1.29 ± 0.09
5BF_S	158.0 ± 5.4	11.7 ± 0.4	49.2 ± 0.6	1.42 ± 0.10
5CF	191.1 ± 4.9	15.7 ± 2.6	62.7 ± 1.7	1.96 ± 0.07
5CF_S	193.3 ± 4.6	16.3 ± 1.4	82.1 ± 1.1	2.82 ± 0.06

**Table 6:** Parameters determined in the accelerated fatigue method.

Index	Max Strength, (kN)	Number of Cycles to Break	$\sigma_{n-1}$ (MPa)	$\sigma_{n}$ (MPa)	$\sigma_{f}$ (MPa)	$Z_{z}$ (MPa)	U(MPa)
PA	2.10	13,590	49.0	52.5	45.5	27.5	61.8
PA_S	2.10	13,790	49.0	52.5	45.5	31.5	62.2
5AF	2.25	14,400	52.5	56.3	48.7	29.9	70.4
5AF_S	2.10	13,600	49.0	52.5	45.5	31.5	69.3
10AF	2.10	13,800	49.0	52.5	45.5	28.0	70.0
10AF_S	2.39	15,460	56.3	59.8	52.8	31.5	71.2
5BF	2.25	14,480	52.5	56.3	48.7	38.5	65.5
5BF_S	2.10	13,690	49.0	52.5	45.5	35.0	67.0
10BF	2.39	15,780	56.3	59.8	52.8	41.3	66.4
10BF_S	2.39	16,000	56.3	59.8	52.8	45.0	75.7
5CF	2.81	18,490	66.8	70.3	63.3	42.5	101.6
5CF_S	2.81	18,790	66.8	70.3	63.3	46.8	110.9
10CF	3.23	21,770	77.3	81.3	73.3	49.0	107.6
10CF_S	3.51	23,530	84.3	87.8	80.8	56.3	114.1

## Data Availability

The raw data supporting the conclusions of this article will be made available by the authors on request.
